# Therapeutic hypothermia after nonshockable cardiac arrest: the HYPERION multicenter, randomized, controlled, assessor-blinded, superiority trial

**DOI:** 10.1186/s13049-015-0103-5

**Published:** 2015-03-07

**Authors:** Jean Baptiste Lascarrou, Ferhat Meziani, Amélie Le Gouge, Thierry Boulain, Jérôme Bousser, Guillaume Belliard, Pierre Asfar, Jean Pierre Frat, Pierre François Dequin, Jean Paul Gouello, Arnaud Delahaye, Ali Ait Hssain, Jean Charles Chakarian, Nicolas Pichon, Arnaud Desachy, Fréderic Bellec, Didier Thevenin, Jean Pierre Quenot, Michel Sirodot, François Labadie, Gaétan Plantefeve, Dominique Vivier, Patrick Girardie, Bruno Giraudeau, Jean Reignier, for the Clinical Research in Intensive Care and Sepsis (CRICS) Group and the HYPERION Study Group

**Affiliations:** Medical-Surgical Intensive Care Unit, District Hospital Center, La Roche-sur-Yon, France; Medical Intensive Care Unit, University Hospital Center, University of Strasbourg, Strasbourg, France; INSERM CIC1415, CHRU de Tours, Tours, France; Université François-Rabelais de Tours, PRES Centre-Val de Loire Université, Tours, France; Medical Intensive Care Unit, Regional Hospital Center, Orleans, France; Medical-Surgical intensive Care Unit, General Hospital Center, Saint Brieuc, France; Medical Intensive Care Unit, South Brittany General Hospital Center, Lorient, France; Medical Intensive Care Unit, University Hospital Center, Angers, France; Medical Intensive Care Unit, University Hospital Center, Poitiers, France; Medical Intensive Care Unit, University Hospital Center, Tours, France; Medical-Surgical Intensive Care Unit, General Hospital Center, Saint Malo, France; Medical-Surgical Intensive Care Unit, General Hospital Center, Rodez, France; Medical Intensive Care Unit, University Hospital Center, Clermond-Ferrand, France; Medical-Surgical Intensive Care Unit, General Hospital Center, Roanne, France; Medical-Surgical Intensive Care Unit, University Hospital Center, Limoges, France; Medical-Surgical Intensive Care Unit, General Hospital Center, Angouleme, France; Medical-Surgical Intensive Care Unit, General Hospital Center, Montauban, France; Medical-Surgical Intensive Care Unit, General Hospital Center, Lens, France; Medical Intensive Care Unit, University Hospital Center, Dijon, France; Medical-Surgical Intensive Care Unit, General Hospital Center, Annecy, France; Medical-Surgical Intensive Care Unit, General Hospital Center, Saint Nazaire, France; Medical-Surgical Intensive Care Unit, General Hospital Center, Argenteuil, France; Medical-Surgical Intensive Care Unit, General Hospital Center, Le Mans, France; Medical Intensive Care Unit, University Hospital Center, Lille, France

**Keywords:** Cardiac arrest, Therapeutic hypothermia, Nonshockable rhythm

## Abstract

**Background:**

Meta-analyses of nonrandomized studies have provided conflicting data on therapeutic hypothermia, or targeted temperature management (TTM), at 33°C in patients successfully resuscitated after nonshockable cardiac arrest. Nevertheless, the latest recommendations issued by the International Liaison Committee on Resuscitation and by the European Resuscitation Council recommend therapeutic hypothermia. New data are available on the adverse effects of therapeutic hypothermia, notably infectious complications. The risk/benefit ratio of therapeutic hypothermia after nonshockable cardiac arrest is unclear.

**Methods:**

HYPERION is a multicenter (22 French ICUs) trial with blinded outcome assessment in which 584 patients with successfully resuscitated nonshockable cardiac arrest are allocated at random to either TTM between 32.5 and 33.5°C (therapeutic hypothermia) or TTM between 36.5 and 37.5°C (therapeutic normothermia) for 24 hours. Both groups are managed with therapeutic normothermia for the next 24 hours. TTM is achieved using locally available equipment. The primary outcome is day-90 neurological status assessed by the Cerebral Performance Categories (CPC) Scale with dichotomization of the results (1 + 2 versus 3 + 4 + 5). The primary outcome is assessed by a blinded psychologist during a semi-structured telephone interview of the patient or next of kin. Secondary outcomes are day-90 mortality, hospital mortality, severe adverse events, infections, and neurocognitive performance. The planned sample size of 584 patients will enable us to detect a 9% absolute difference in day-90 neurological status with 80% power, assuming a 14% event rate in the control group and a two-sided Type 1 error rate of 4.9%. Two interim analyses will be performed, after inclusion of 200 and 400 patients, respectively.

**Discussion:**

The HYPERION trial is a multicenter, randomized, controlled, assessor-blinded, superiority trial that may provide an answer to an issue of everyday relevance, namely, whether TTM is beneficial in comatose patients resuscitated after nonshockable cardiac arrest. Furthermore, it will provide new data on the tolerance and adverse events (especially infectious complications) of TTM at 32.5-33.5°C.

**Trial registration:**

ClinicalTrials.gov: NCT01994772.

This manuscript was written in accordance with SPIRIT guidelines [1].

## Introduction

### Background and rationale

Cardiac arrest remains a major cause of mortality, as well as a cause of disability in survivors [2]. In Europe, 300 000 cardiac arrests occur annually, of which 250 000 are fatal. Even among patients with good prognostic factors, less than 50% are discharged from the hospital without severe neurological impairments [3,4].

After early work suggesting a neuroprotective effect of hypothermia [5], animal studies provided evidence of neurological recovery after a period of controlled hypothermia [6]. These findings were confirmed by two randomized trials performed 10 years ago and published in the same issue of the *New England Journal of Medicine*. In both trials, therapeutic hypothermia significantly improved the neurological outcomes of patients with cardiac arrest in shockable rhythms (ventricular fibrillation or pulseless ventricular tachycardia). The first randomized trial was a European multicenter study in 275 patients managed with normothermia or hypothermia [7]. After 6 months, the proportion of patients with good neurological outcomes was 55% with hypothermia compared to only 39% with normothermia (odds ratio [OR], 1.4; *P* = 0.009). In addition, mortality was significantly lower in the hypothermia group (OR, 0.74; *P* = 0.02). The second randomized trial was an Australian multicenter study in 77 patients managed with normothermia or hypothermia [8]. Again, good neurological outcomes were more common in the hypothermia group (OR, 1.88; *P* = 0.046), and this difference persisted in the multivariate analysis (OR, 5.25; *P* = 0.011). However, hypothermia was not associated with a significant decrease in mortality (OR, 1.75; *P* = 0.145). A 2012 Cochrane review and metaanalysis confirmed the neuroprotective effect of hypothermia (relative risk [RR] of achieving a good neurological outcome, 1.55; 95% confidence interval [95% CI], 1.22-1.96) [9].

These data firmly establish the efficacy of TTM between 32°C and 34°C in improving the neurological outcomes of patients with shockable cardiac arrest. Guidelines issued in 2010 by the European Resuscitation Council (ERC) [10] and International Liaison Committee on Resuscitation (ILCOR) [11] recommend the routine use of TTM between 32°C and 34°C in comatose survivors of cardiac arrest with a nonshockable rhythm at arrival of emergency-care providers. These recommendations are based on a low level of evidence (Class IIb, LOE B). No randomized trials have assessed therapeutic hypothermia in survivors of nonshockable cardiac arrest. The recommendations are based on retrospective cohort studies and on prospective studies evaluating the feasibility of achieving a period of 32°-34° TTM using invasive [12] or semi-invasive [13] devices. Nevertheless, the results of these studies are conflicting.

- A metaanalysis [14] showed no significant improvement in the 6-month neurological outcomes of 897 patients included in retrospective cohorts (OR, 0.93; 95% CI, 0.88-1). In addition, two recent prospective cohort studies not included in the meta-analysis -- one from France published in *Circulation* [15] and the other from Finland published in *Intensive Care Medicine* [16] -- failed to demonstrate benefits from 32°-34° TTM in survivors of nonshockable cardiac arrest. Two German studies [17,18], one retrospective and the other prospective, showed no decrease in mortality when 32°-34° TTM was combined with optimal post-cardiac arrest care (including prompt coronary angiography if appropriate and hemodynamic parameter optimization). A very recent post-hoc analysis of the randomized TTM Study [19] showed no difference in neurological outcomes in the 178 patients with nonshockable rhythms [20].
- Other studies exhibiting similar methodological weaknesses (retrospective or prospective nonrandomized design without blinded assessment), in contrast, suggest a beneficial effect of hypothermia. Two retrospective Austrian studies showed better neurological outcomes with 32°-34° TTM in 374 patients [21] and 828 patients [22], respectively, with shockable or nonshockable cardiac arrest. Finally, a case–control study of 100 patients with nonshockable cardiac arrest reported by an American group showed better neurological outcomes with 32°-34° TTM [23].

Three factors probably explain most of the difference in 32°-34° TTM effects between patients with shockable versus nonshockable cardiac arrest. First, patients with shockable cardiac arrest constitute a fairly uniform population of predominantly male individuals with a cardiac cause (ST-segment-elevation myocardial infarction) potentially treatable by coronary angiography and appropriate percutaneous coronary intervention [24]. In contrast, nonshockable cardiac arrest can occur in a wide variety of settings, for instance as a complication of a primary shockable rhythm or during asphyxia due to hanging, drowning, or gastric-content aspiration. Second, nonshockable cardiac arrest carries a poorer prognosis than does shockable cardiac arrest: survival with good neurological function (CPC 1 or 2) is nearly 40% after shockable cardiac arrest compared to less than 20% after nonshockable cardiac arrest [25,26]. Two factors associated with a better prognosis of cardiac arrest are less common in nonshockable than shockable rhythms, namely, presence of a witness [27] and cardiac origin of the arrest [28]. Consequently, demonstrating a beneficial effect of a therapeutic intervention requires a larger sample size in studies of nonshockable compared to shockable cardiac arrest. Third, a corollary to the first and second factors is that the effects of interventions for cardiac arrest may differ markedly between patients with nonshockable and shockable cardiac arrest. For example, although recent data argue against epinephrine treatment during shockable cardiac arrest [29,30], other studies strongly support the earliest possible administration of epinephrine during nonshockable cardiac arrest [31].

Studies of neonatal hypoxia have produced convincing evidence that 32°-34° TTM diminishes mortality and improves neurological outcomes [32,33]. Although brain plasticity differs between neonates and adults, this evidence strongly supports a beneficial effect of 32°-34° TTM in patients with cardiac arrest due only to anoxia. A retrospective study showed that the mortality rate after attempted suicide by hanging with cardiac arrest was as high as 90% [34]. In contrast, in two studies of 32°-34° TTM used to treat patients with severe asphyxia, mortality was less than 67% [35] and 45% [36], respectively. These findings support evaluation of 32°-34° TTM in patients with nonshockable cardiac arrest due to noncardiac causes [37].

The safety profile of 32°-34° TTM was good in two early studies [7,8]. More recently, however, an increased risk of infection [38], including pneumonia [39] was reported. In addition, 32°-34° TTM usually requires neuromuscular blockade, which interferes with the neurological assessments, thereby extending ICU stay length and diminishing the ability to identify seizures and to provide optimal seizure prevention [40]. Activation of the inflammatory cascade may occur during 32°-34° TTM [41], leading to a risk of rebound hyperthermia, which may adversely affect patient outcomes [42,43]. Similarly, 32°-34° TTM may exert negative hemodynamic effects, increasing vasoactive drug requirements [44], a point of particular concern given the poor prognosis of post-resuscitation shock [16].

In sum, the risk/benefit ratio of 32°-34° TTM in patients with nonshockable cardiac arrest remains unclear. This uncertainty has led to a call for randomized controlled trials [45].

### Objectives

#### Primary objective

To determine whether TTM between 32.5°C and 33.5°C (32.5°-33.5° TTM) for 24 h improves day-90 neurological outcomes compared to TTM between 36.5°C and 37.5°C in survivors of nonshockable cardiac arrest.

#### Secondary objectives

To determine whether 32.5°-33.5° TTM for 24 h decreases mortality and morbidity (ICU and hospital stay lengths) and to assess the safety of 32.5°-33.5° TTM.

### Trial design

HYPERION is a multicenter, randomized, controlled, assessor-blinded, superiority trial with two parallel groups and a primary endpoint of day-90 neurological outcome.

## Methods: participants, interventions, and outcomes

### Study setting

The HYPERION trial is taking place in 22 ICUs in 22 hospitals (8 university and 14 general hospitals) in France.

### Eligibility criteria

**Figure 1:**
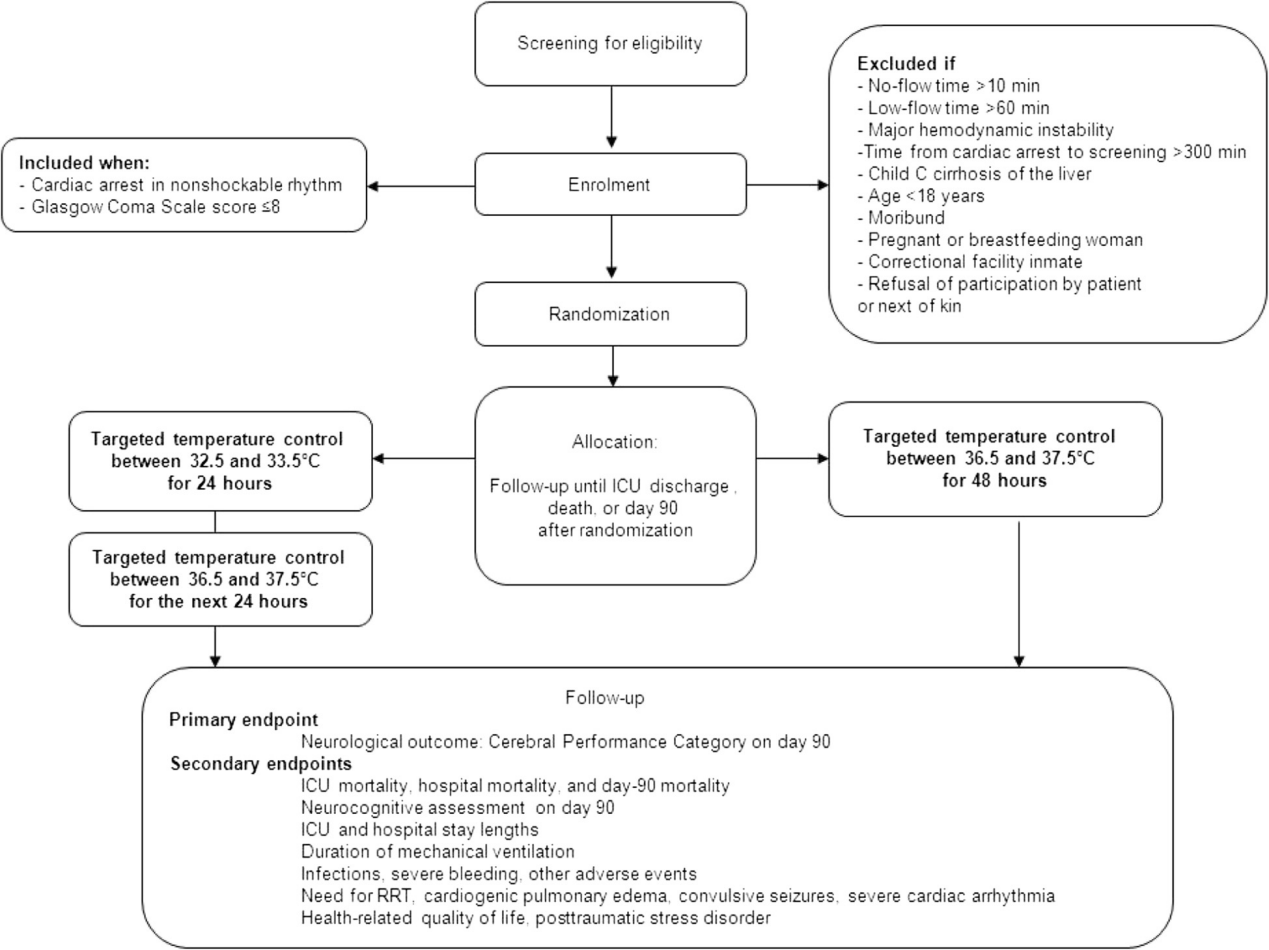
**Study flowchart.** ICU, intensive care unit; RRT, renal replacement therapy.

Figure 1 shows the patient eligibility criteria and study protocol.

#### Inclusion criteria

Patients must meet both of the following criteria:

- Cardiac arrest in nonshockable rhythm and
- Glasgow Coma Scale score ≤8. In patients receiving sedative therapy at ICU admission, the Glasgow Coma Scale score assessed by the emergency physician just before sedative therapy initiation is used.

#### Exclusion criteria

Patients fulfilling one or more of the following criteria are not included:

- No-flow time >10 min (time from collapse to initiation of external cardiac massage);
- Low-flow time >60 min (time from initiation of external cardiac massage to return of spontaneous circulation).
- Major hemodynamic instability (defined as a continuous epinephrine or norepinephrine infusion at a flow rate >1 μg/Kg/min)
- Time from cardiac arrest to study inclusion >300 min
- Moribund patient
- Child C cirrhosis of the liver
- Age <18 years
- Pregnant or breastfeeding woman
- Correctional facility inmate
- Previous inclusion in another randomized clinical trial on cardiac arrest with day-90 neurological outcome as the primary endpoint
- Patient without health insurance
- Decision by the patient or next of kin to refuse the study.

### Interventions

**Figure 2:**
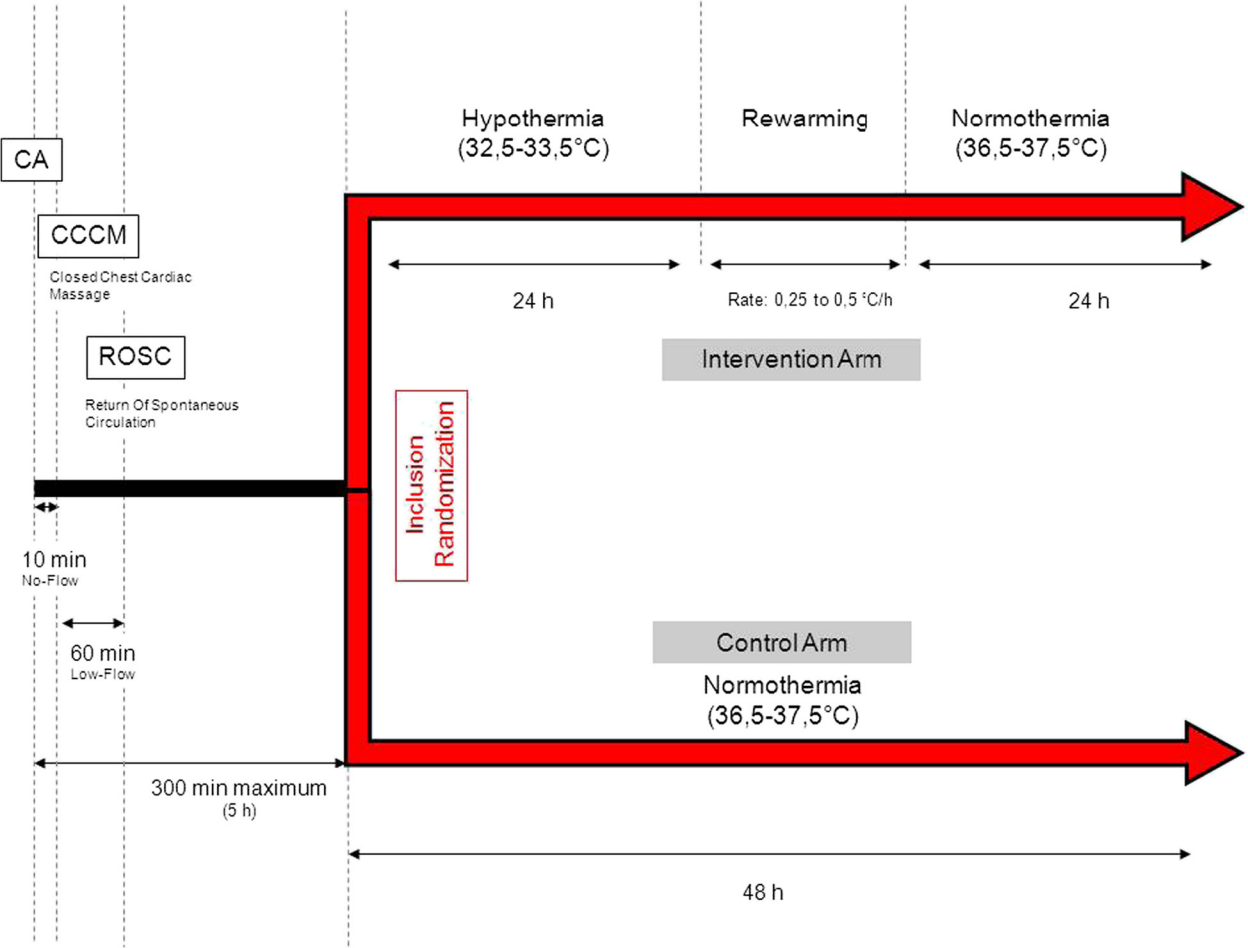
**Inclusion criteria related to the cardiac arrest and study procedures.** CA, cardiac arrest; CCCM, closed chest cardiac massage; ROSC, return of spontaneous circulation.

Patients allocated at random to 32.5°-33.5° TTM have hypothermia induced then maintained for 24 h. Slow rewarming to 36.5°-37.5° is then achieved and maintained for 24 h. Patients in the control group have their body temperature maintained at 36.5°-37.5°C for 48 h. Figure 2 provides details on the study protocol and randomization arms.

To induce and maintain 32.5°-33.5° TTM, each center follows its standard protocol. Thus, the method may involve active internal cooling using a specific device, active external cooling using a specific device, or active external cooling without a specific device. No trial using mortality or neurological outcome as the endpoint has demonstrated that one method is better than the others [46,47]. Infusion of cold fluid (4°C) is recommended to expedite achievement of the target temperature [48-50]. The same methods are used to manage hyperthermia (above 37.5°C) in both groups. The study protocol involves standardization of several parameters including sedation, neuromuscular blockade, and management of expected adverse events. The use of medications (e.g., acetaminophen or aspirin) to maintain normothermia is discouraged in both groups [51]. Figure 2 recapitulates the therapeutic interventions.

#### Concomitant medications/treatments in both groups

##### Sedation

In the 32.5°-33.5° TTM group, all patients receive sedation with midazolam or propofol combined with fentanyl or sufentanil according to the standard sedation protocol in each participating ICU. Doses are adjusted to obtain a Richmond Agitation Sedation Scale score of −5 [52] and are tapered when body temperature is above 36°C during the rewarming phase.

In the 36.5°-37.5° TTM group, all patients receive sedation with midazolam or propofol combined with fentanyl or sufentanil for the first 12 h after randomization. Doses are adjusted to obtain a Richmond Agitation Sedation Scale score of 0. As indicated in the 2010 ILCOR guidelines [10], no data exist to support the use of sedative agents during TTM at 37°C. The use of sedative agents during TTM at 37°C is therefore restricted, to shorten the time to awakening.

##### Shivering and neuromuscular blockade

Persistent shivering is treated according to a previously published three-step protocol [53] that has been adapted since the publication of the Bedside Shivering Assessment Score (BSAS) [54,55]. The goal is to obtain a BSAS ≤1.

- Step 1, single intravenous bolus of a hypnotic agent and an opioid, in doses equal to the hourly infusion rates of hypnotic and opioid drugs (i.e., 5-mg intravenous midazolam bolus if the continuous midazolam infusion rate was 5 mg/h);
- Step 2, intravenous bolus of a nondepolarizing neuromuscular blocker (i.e., 10 mg of cisatracurium);
- and Step 3, continuous infusion of a nondepolarizing neuromuscular blocker (i.e., cisatracurium in an initial dose of 10 mg/h), with a BSAS target of ≤1; during rewarming, the infusion may be stopped when the core body temperature increases above 35°C.

#### Concomitant prevention of systemic secondary brain injury in both groups

##### Arterial hypotension

Hemodynamic evaluations are conducted to allow blood volume optimization. Hypovolemia is managed with crystalloid or colloid infusion, according to standard practice in the participating ICU. Subsequent evaluations are performed as dictated by the course of the hemodynamic parameters. In accordance with guidelines [10], and in the absence of specific randomized studies, a mean arterial pressure of 65 mmHg and, if measured, central venous oxygen saturation (ScvO_2_) ≥70% are considered reasonable targets [11]. The introduction of vasoactive drug treatment is at the discretion of the physicians, who follow international guidelines [10] and local protocols.

##### Hypoxemia

PaO_2_ and pulse oximetry (SpO_2_) are monitored with the goal of maintaining SpO_2_ ≥ 92%, as recommended [10].

##### Hypercapnia and hypocapnia

PaCO_2_ and end-tidal partial CO_2_ pressure are monitored with the goal of maintaining PaCO_2_ between 35 and 40 mmHg after correcting for body temperature [56].

##### Anemia

The goal is to maintain hemoglobin ≥7 g/dL in patients without ischemic heart disease and ≥10 g/dL in those with ischemic heart disease, as recommended [10].

##### Blood sugar control

A protocol for monitoring blood glucose (or capillary blood glucose) is available to the physician in charge of the patient. In accordance with guidelines [10], specific treatment is recommended in patients with blood glucose values outside the 3.33-10 mmoL/L (60–180 mg/dL) range.

#### Withdrawal of life-sustaining treatments in both groups

Each participating ICU uses its own specific protocol to make decisions about withdrawing life-sustaining treatments. Routine evaluation of the neurological prognosis by an out-of-house consultant is not performed. Nevertheless, to ensure that neurological outcome prediction is performed according to the most recent evidence, our protocol includes the following three measures [57].

1. During the trial preparation phase, all investigators received specific instructions that decisions to withdraw life-sustaining treatments must comply with guidelines issued in 2012 by the ethics committee of the French Intensive Care Society (SLRF) [58]. According to these guidelines, multiple criteria should be used to predict the neurological outcome. Taken individually, the following criteria predict an unfavorable outcome of postanoxic coma after cardiac arrest: bilateral absence of the pupillary response to light or corneal reflex on the third day; bilateral absence of a motor response to pain on the third day; persistent generalized myoclonus during the first 24 hours; an EEG showing an isoelectric line or burst-suppression or anoxic status epilepticus during the first week; and bilateral absence of early cortical activity (N20) detected by somatosensory evoked potentials after the third day and after the end of hypothermia. Although wrongly predicting an unfavorable outcome cannot be entirely avoided, the risk of this occurring can be decreased by relying on more than one criterion. Another important point when seeking to improve prediction accuracy is careful and exhaustive collection of the most severe results from evaluations of each criterion [58].
2. During the trial, the investigators receive newsletters that detail any new information on predicting neurological outcomes after cardiac arrest and any new French or international guidelines about patients with coma after cardiac arrest.
3. The eCRF includes a specific section for collecting the following data in patients with a decision to withdraw life-sustaining treatments: clinical, laboratory, and imaging-study findings on the day the decision was taken; details on the decision-making process; and details on implementation of the decision.

### Outcomes

#### Primary outcome measure

- Neurological status 90 days after randomization according to the CPC Scale [59-61]. The CPC is assessed during a semi-structured telephone interview [62]. All interviews of study patients are performed by a single psychologist specifically trained for the study and blinded to the treatment group.

#### Secondary outcome measures

- ICU mortality
- Hospital mortality
- Day-90 mortality
- Day-90 quality of life evaluated using the 36-item Short Form for Health Survey [63,64]
- Day-90 patient self-sufficiency evaluated using the Activities of Daily Living (ADL) Index [65,66], modified Barthel’s ADL index [67], and two simple standardized questions [68]
- Day-90 neurocognitive status evaluated using the telephone version of the Mini-Mental State Examination [69]
- Estimated number of patients with symptoms of posttraumatic stress on day 90 as assessed using the revised Impact of Events Scale (IES) [70].
- ICU stay length
- Duration of mechanical ventilation, defined as the time from randomization to final successful extubation. Extubation followed by breathing without invasive ventilatory assistance (not including noninvasive ventilation) for 48 h is classified as successful [71].
- Incidence of severe bleeding defined as red-blood-cell transfusion or surgery for intracranial hemorrhage
- Incidence of nosocomial infections in the ICU
- Incidence of aspiration pneumonia in the ICU, with aspiration pneumonia defined as pneumonia diagnosed within 48 h of mechanical ventilation initiation
- Incidence of ventilator-acquired pneumonia in the ICU
- Incidence of nosocomial urinary tract infections in the ICU
- Incidence of intravascular catheter infections in the ICU
- Need for vasoactive drug therapy within the first 48 h
- Need for renal replacement therapy
- Number of episodes of cardiogenic pulmonary edema
- Number of episodes of seizures
- Number of episodes of severe cardiac arrhythmia.

### Participant timeline

Participant timeline is figured on Table 1.

**Table 1:** **Participant timeline**

	**Inclusion**	**D0**	**D1**	**D2**	**D3**	**D4**	**Dn**	**D90**
Eligibility: check inclusion and exclusion criteria	X							
Informed consent	X							
Demographic data	X							
Randomization	X							
Patient characteristics and Physical examination	X							
Vital signs	X							
Coronary angiography	X						X	
Mechanical ventilation		X	X	X	X	X	X	
Biology		X	X	X	X	X	X	
Electrocardiogram	X		X	X	X			
Physical examination	X		X	X	X	X	X	
Treatments		X	X	X	X	X	X	
Vital status		X	X	X	X	X	X	X
Telephone interview by psychologist								X

Dn: Each participant is followed until day 7 or ICU discharge, whichever occurs first.

### Sample size

We expect that 14% of patients in the 36.5°-37.5° TTM group will be CPC 1 or 2 on day 90 and that this proportion will increase by 9% with 32.5°-33.5° TTM. With a two-sided Type I error of 5% and 80% power, and given that two interim analyses will be performed, the required number of patients is 292 per group, i.e., 584 patients in all.

### Recruitment

Patient inclusion started in January 2014 in 22 French ICUs. Enrolment is ongoing. As of February 2, 2015, 161 patients had been included.

## Methods: assignment of interventions

### Allocation

Randomization is centralized, web-based, and accessible 24 hours a day. Randomization is balanced (1:1) and stratified by center and cause of cardiac arrest (probable cardiac cause such as cardiac ischemia or probable noncardiac cause).

### Sequence generation

The randomization sequence was generated by a statistician from the INSERM CIC 1415 not involved in patient recruitment. The sequences are implemented in the software used to collect the data (eCRF).

### Blinding

Blinding of healthcare workers, patients (despite the sedation), and families to the type of temperature management is not feasible. However, the primary outcome is assessed by a psychologist blinded to the treatment arm, during a semi-structured telephone interview [62].

## Methods: data collection, management, and analysis

### Data collection and management

The study data are recorded in an electronic web-based case-report form (eCRF) from the medical records of each patient (source data), by trial-site personnel. The data manager, in cooperation with the coordinating investigator, establishes the trial database by exporting data from the eCRF. Any protocol deviations are recorded in either the eCRF or the medical records.

### Statistical methods

#### Statistical analysis

A predefined statistical analysis plan will be followed. The intention-to-treat principle will be applied. The statistical report will incorporate the data recommended by the CONSORT Statement extension for nonpharmacologic treatment interventions [72].

Descriptive statistics will be used to compare the two randomization groups, without statistical testing. The chi-squared test will be performed to compare the primary endpoint in the two groups. Two interim analyses will be conducted, after the inclusion of 200 and 400 patients, respectively. The rule developed by Peto and Haybittle [73] will be applied, with the significance level set at 0.001 for both interim analyses and the significance level associated with the final analysis set at 0.049 to maintain an overall Type I error of 5%.

Secondary endpoints evaluating binary variables (need for renal replacement therapy, acute cardiogenic pulmonary edema, and binary day-90 neurological outcome) will be compared using the chi-squared test. Secondary endpoints evaluating quantitative variables (biological tests, stay lengths, and mechanical ventilation duration) will be compared using the Student t test or Mann–Whitney test.

#### Subgroup analysis

- Based on a previous trial showing that benefits from 33°C TTM in cardiac arrest were greatest in the subgroups of patients with long no-flow times and low-flow times (most notably longer than 15 minutes) [74-76], we plan to perform analyses in these two subgroups.
- We will also analyze other subgroups defined based on the following prognostic factors: presence of a witness (yes/no); in-hospital versus out-of-hospital cardiac arrest; and probable cardiac origin (yes/no), which was a stratification variable.
- Finally, to take the baseline cerebral performance category into account, we will compare subgroups with CPC1-2 versus CPC 3–4 before randomization.

## Methods: monitoring

### Data monitoring

The results of the interim analysis will be provided to the Data and Safety Monitoring Board (DSMB), which will make recommendations about whether to continue or stop the trial. The DSMB is composed of 3 physicians not otherwise involved in the trial. For both interim analyses, the DSMB will have access to unblinded results on day-90 CPC, day-90 mortality, and secondary safety outcomes (dialysis, infection, and seizure event). The results of the interim analysis will not be disclosed unless they lead the DSMB to request premature trial discontinuation. The DSMB members are Prof. Pierre Francois Laterre (Department of Critical Care Medicine, Cliniques Universitaires Saint Luc, Université Catholique de Louvain, Avenue Hippocrate, 10, 1200 Brussels, Belgium), Prof. Eric Maury (Medical ICU, Saint-Antoine Teaching Hospital, Assistance Publique-Hôpitaux de Paris, 184 rue du Faubourg Saint-Antoine, Cedex 12, Paris 75571, France), and Prof. Bruno Megarbane (Medical ICU, Lariboisière Teaching Hospital, Paris-Diderot University, Paris, France).

### Harms

The trial may be temporarily stopped for an individual patient, at the discretion of the attending physician, in case of major serious adverse events suspected to be associated with the type of temperature management. According to French law, as the strategies used in both study arms are classified as standard care, no specific reporting procedure for unexpected serious adverse events is planned. Follow-up data on expected serious adverse events are recorded in the eCRF; these events may include bleeding, cardiogenic pulmonary edema, renal replacement therapy, infection, and withholding or withdrawing of life-sustaining treatments.

## Ethics and dissemination

### Research ethics approval

The trial is conducted in compliance with the current version of the Helsinki Declaration and good clinical practice guidelines. The research project was approved by the ethics committee of the French Intensive Care Society (SRLF) on October 6, 2011, and by the appropriate Ethics Committee for the Protection of Patients (CPP Ouest 2) in Angers, France, on June 11, 2013.

### Consent or assent

According to French law, because the strategies used in both study groups are considered components of standard care, there is no requirement for *consent*. Instead, *information* of the patients or next of kin is required. All patients assessed for enrolment in the trial are comatose and therefore unable to understand information. Their next of kin is informed about the trial and confirms in writing that he/she has received this information. If no next of kin can be contacted during screening for the study, trial inclusion is conducted as an emergency procedure by the ICU physician, in compliance with French law. Patients who regain consciousness are informed about the trial as soon as possible and asked for written confirmation that they received this information.

A patient may withdraw from the trial at any time if the person informed about the study (patient or next of kin) is unwilling to continue in the trial. The person requesting trial withdrawal is asked for permission to continue data recording and to perform the part of the day-90 telephone interview consisting in a semi-structured CPC assessment; patients who accept this partial withdrawal modality have their data kept in the study analyses. Patients who request full withdrawal have all their data deleted.

Patients transferred to another ICU are withdrawn from the trial if the transfer occurs before the end of the TTM phase, unless the new ICU is participating in the trial, in which case the patient is kept in the same treatment group. All patients transferred to another ICU are followed for determination of the primary endpoint.

### Confidentiality

The study data will be handled as requested by the French Data Protection Authority (Commission Nationale de l’Informatique et des Libertés). All original records will be kept on file at the trial sites or the coordinating data managing center for 15 years. The clean electronic trial database file will be anonymized and kept on file for 15 years.

## Discussion

**Figure 3:**
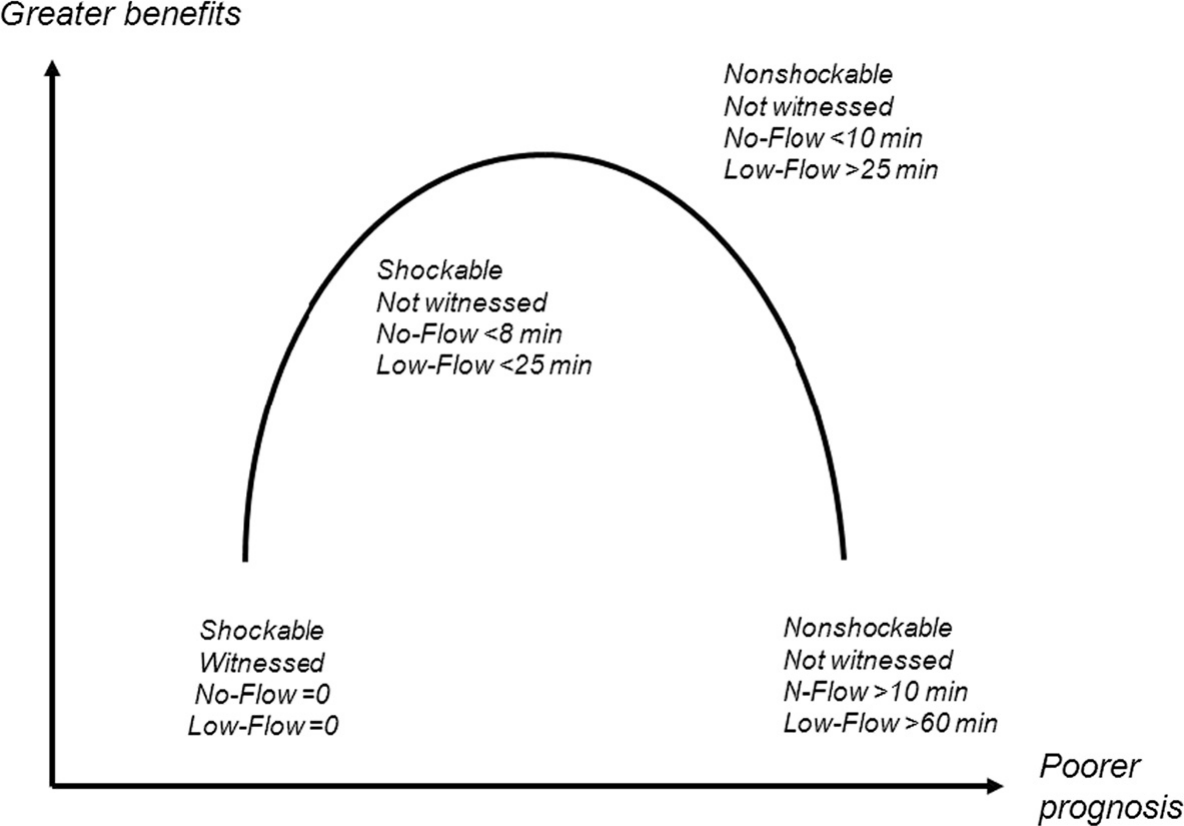
**Benefits from targeted temperature management in relation to patient’s prognosis.**

**Figure 4:**
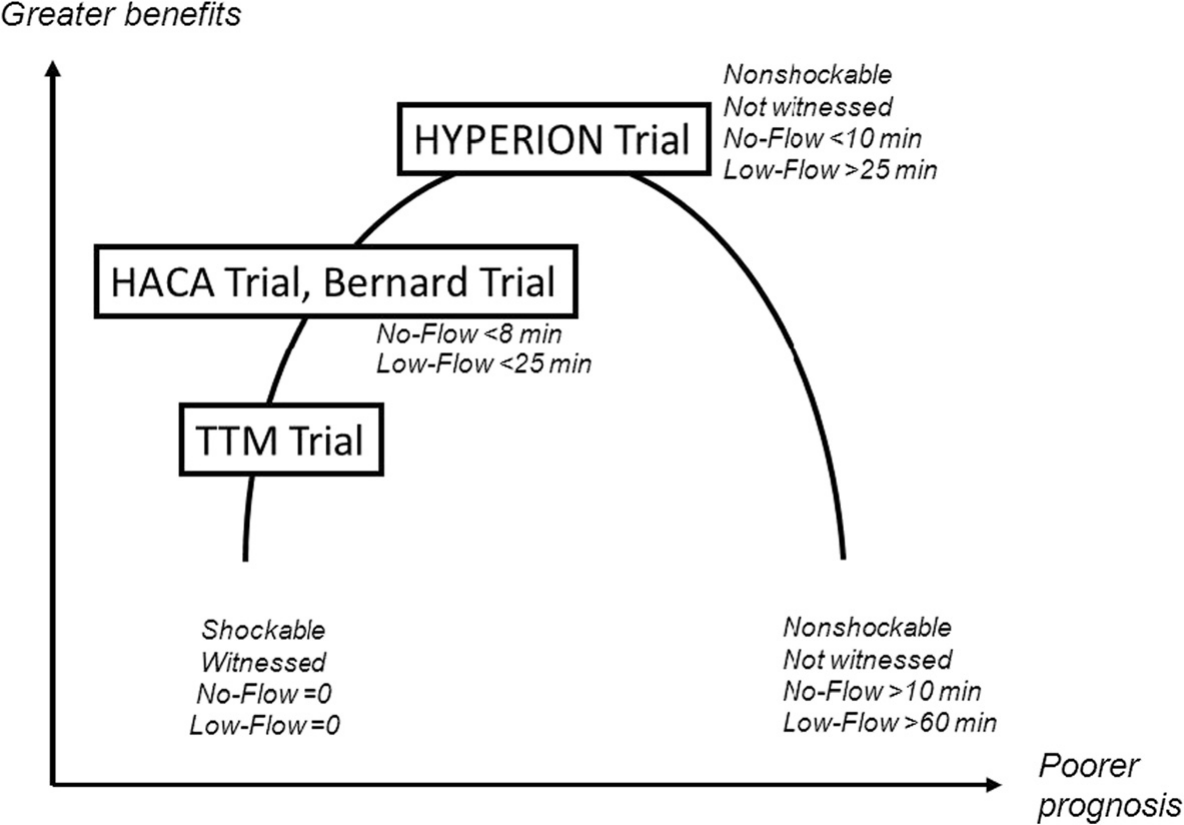
**Benefits from targeted temperature management in relation to patient’s prognosis documented in previous studies and expected in the HYPERION trial.**

Few data are available to explain the discrepancies among the findings from trials of TTM, most notably in patients with nonshockable cardiac arrest. The beneficial effects of 32.5°-33.5° TTM probably vary with the degree of brain damage at treatment initiation. The ischemic penumbra concept developed for stroke is probably applicable to the brain damage seen after cardiac arrest. In a study of patients with nonshockable cardiac arrest, the benefits from 32.5°-33.5° TTM were greatest when the no-flow time was longer than 8 minutes [76]. Similarly, in another study of patients with shockable or nonshockable cardiac arrest, 32.5°-33.5° TTM was most effective in the subgroup whose time from cardiac arrest to the return of spontaneous circulation (ROSC) was longer than 15 minutes [74]. In a previous pilot study, 32° was better than 34° as the target temperature [77]. The TTM Trial published in *The New England Journal Medicine* [19] showed that TTM at 36°C was similar to TTM at 33°C in patients with good prognostic factors including witnessed cardiac arrest in shockable rhythm with short no-flow and low-flow times compared to patients enrolled in earlier trials and having less favorable prognostic characteristics [7,8]. Figure 3 reports the estimated benefits of TTM and Figure 4 the effects documented in previous studies and the expected effects in the HYPERION trial.

Our decision to use a 24-h period of normothermia after 32.5°-33.5° TTM was based on reports that rebound hyperthermia adversely affected the outcomes of patients with cardiac arrest [42,78]. Whether rebound hyperthermia is a causal or an associated factor for mortality or neurological outcome after 32.5°-33.5° TTM is unclear. However, the TTM Trial [19] established the importance of preventing hyperthermia after cardiac arrest.

In conclusion, the HYPERION trial is the first registered randomized controlled trial evaluating the potential benefits of 32.5°-33.5° TTM on the neurological outcome of patients after nonshockable cardiac arrest.

## Abbreviations

BSAS: Bedside shivering assessment scale
CPC: Cerebral performance category
eCRF: Electronic case-report form
ICU: Intensive care unit
ROSC: Return of spontaneous circulation
TTM: Targeted temperature management
